# An Insight into the Presence of Antimicrobial Resistance Genes in Opportunistic Pathogenic Bacteria Isolated from Farm-Reared Crickets

**DOI:** 10.3390/microorganisms13020391

**Published:** 2025-02-11

**Authors:** Teresita d. J. Bello Gonzalez, Betty van Gelderen, Frank Harders, Alex Bossers, Michael S. M. Brouwer, Olga L. M. Haenen

**Affiliations:** 1 Department of Bacteriology, Host-Pathogen Interaction and Diagnostic Development, Antimicrobial Resistance Group, Wageningen Bioveterinary Research, Wageningen University Research, P.O. Box 65, 8200 AB Lelystad, The Netherlands; mike.brouwer@wur.nl; 2 National Reference Laboratory for Fish Diseases, Wageningen Bioveterinary Research, Wageningen University Research, P.O. Box 65, 8200 AB Lelystad, The Netherlands; betty.vangelderen@wur.nl (B.v.G.);; 3 Department of Epidemiology, Bioinformatics and Animal Models, Wageningen Bioveterinary Research, Wageningen University Research, P.O. Box 65, 8200 AB Lelystad, The Netherlands; frank.harders@wur.nl (F.H.); or a.bossers@uu.nl (A.B.); 4 Department of Population Health Sciences, Institute for Risk Assessment Sciences, Utrecht University, 3584 CM Utrecht, The Netherlands

**Keywords:** edible insects, crickets, culturable bacteria, whole genome sequencing, antibiotic resistance, microbial safety

## Abstract

To support the role of insects as sustainable feed and food ingredients, evaluating their potential microbiological risk and safety is crucial. In this study, we investigated the presence of antimicrobial resistance (AMR) genes in selected live opportunistic pathogenic bacteria isolated during the rearing process from clinically healthy farm-reared crickets. Molecular analysis was performed by wholegenome sequencing of a total of 14 of these bacterial strains, 7 from house crickets (*Acheta domesticus*) and 7 from banded crickets (*Gryllodes sigillatus*), belonging to *Enterobacteriaceae*, *Staphylococcaceae*, *Enterococcaceae*, and *Bacillaceae* families. The β-lactam AMR genes (*bla*_OXY2-6_, *bla*_ACT-16_, and *bla*_SHV_ variants) were the most predominant genes identified, mainly in *Enterobacteriaceae* strains and in association with fosfomycin (*fos*A) and *oqx*AB efflux pump complexes. In addition, *bla*Z and *mec*A genes were detected in *Bacillus cereus* and *Mammaliicoccus sciuri* strains isolated from both insect species. Genetic mobile elements including IncFIA, IncFIB, IncHI1A, IncHI1B, rep13, and Col3M-like plasmids were detected in *Klebsiella pneumoniae*, *Enterobacter hormaechei*, *Staphylococcus arlettae*, and *B. cereus*, respectively. The results indicate that, not only in the final product but also during the insect-rearing process, microbial safety control, regarding the presence of pathogenic bacteria and AMR genes, is essential for effectively decreasing the microbiological risk between cricket batches within their environment and in terms of the related feed and food chain.

## 1. Introduction

The consumption of insects as food has been part of the culture and traditional practices in many regions worldwide [1]. According to the United Nations, the global population is expected to increase to 9.8 billion by 2050 [2]. Therefore, there is an increasing demand for proteins in the food production chain and a search for alternative protein sources. To satisfy this growing demand for protein, insects have been proposed as an alternative protein source for food and feed [3,4]. Several studies showed that insects have enormous nutritional values, including high levels of proteins, essential amino acids, unsaturated fatty acids, fiber, micronutrients, and vitamins [5]. An insect’s nutritional value depends on several factors including the insect species, developmental stage, rearing conditions, and the substrate used as insect feed [4]. Moreover, it has been demonstrated that, during the insect-rearing process, low greenhouse gas emissions occur and low water use and a small land area are required, providing a low environmental impact compared to that of the livestock industry. Likewise, insects are thought to have a higher feed conversion efficiency by converting plant proteins into insect proteins better than other animals do and to grow on organic waste, which may reduce the costs of the rearing process [6,7].

In 2015, the European Commission (EC) included whole insects and insect-based foods as “novel food”, according to the Commission-Regulation within the European Union (EU) 2015/2283 [8]. Moreover, in 2021, the EU adopted the Commission-Regulation (EU) 2021/1925 amending Regulation (EU) 142/2011, in which the use of processed animal proteins (PAPs) from insects, such as insect meal, is authorized in animal feed. Within the list of insect species authorized in the EU for the production of PAPs intended as feed for farm animals, three species of crickets, *Gryllodes sigillatus* (banded cricket), *Gryllus assimilis* (Jamaican field cricket), and *Acheta domesticus* (house cricket), are included due to their high protein and fat content levels [9,10]. In 2023, the EC allowed *A. domesticus* (house cricket) partially defatted powder as a novel food in food products according to (EU) 2023/5, thereby amending Implementing Regulation (EU) 2017/2470 [11,12].

Crickets are members of the Orthoptera order and are recognized as one of the most common types of edible insects consumed as food worldwide [13]. An increasing number of cricket farms for food and feed has been established as a result of the low environmental impact, sustainability, nutritional source, and nutraceutical properties of crickets [14,15]. Likewise, the scientific community is continuously exploring the health benefits of crickets, including their nutritional values as well as the potential microbiological risks associated with bacterial contamination with opportunistic pathogens and antibiotic usage during the mass-rearing process since insects can act as vectors for the transmission of pathogens to humans, animals, or plants [16].

The microbial community present in crickets is dominated by several members of the phyla Pseudomonadota, Bacillota, and Bacteriodota [17]. It can be influenced by the intrinsic microbiome of the insect itself, e.g., the microbial community residing in the gut, and by the microbial diversity introduced during the breeding process including the vertical transmission from mother to offspring [18], rearing process conditions, handling, hygiene, environmental conditions, and substrate used as feed [19]. Together, all these factors have an impact in the insect microbial communities, microbial counts, and the presence of potential opportunistic pathogenic bacteria, including harbored antibiotic resistance genes. The presence of these microbiological risks suggests the need for a critical food safety measurement to ensure a decrease in the levels of contaminants during the rearing process in edible insects used for food and feed.

Antimicrobial resistance (AMR) is considered a public human and animal health concern, particularly in the animal food production chain since food represents one of the main sources for the acquisition, persistence, and spread of antibiotic-resistant microbes and their associated genes into the human gut [20]. In this context, the microbiological safety concerns on an insect-rearing farm increase if bacteria carrying AMR genes are present, due to their potential to be transmitted to other bacteria through genetic mobile elements such as plasmids. This highlights the need for frequent microbiological monitoring on insect-rearing farms during the entire rearing process to prevent and act on the presence of biological hazards within the insect farm, as advised in the Netherlands by the Council of Animal Affairs (RDA) in 2018 [21]. Several reports on the presence of microbial contaminants on and in edible crickets during processing do not consider the presence of dominant bacteria and their potential repertoire of AMR genes during the rearing process, i.e., before harvesting, underestimating the potential of entomopathogens and opportunistic pathogenic bacteria to perpetuate during the insect production cycle and thereafter in the food chain [22,23]. Insects can serve as a reservoir of AMR genes [24]. Previous studies have focused on the identification of AMR genes in crickets [25,26] through polymerase chain reaction (PCR) assays targeting encoding genes for resistance to conventional antibiotics used in clinical practices in which the most predominant genes investigated belonged to the β-lactams, tetracycline, and macrolide antibiotic classes, all detected directly from the insects. This approach limited the spectrum of AMR genes and most importantly the potential presence of genetic mobile elements that could facilitate the spread of these AMR genes between bacteria.

Due to the limited available data on edible crickets regarding the presence of entomopathogens and potential opportunistic pathogenic bacteria, data on their AMR genes repertoire during the insect-rearing process, particularly those associated with genetic mobile elements, are lacking so far. The present study was conducted to gain more insight into the presence of AMR genes in a group of selected live opportunistic pathogenic bacteria with potential biological risk, isolated from clinically healthy farmed house and banded crickets, by using a Whole Genome Sequencing (WGS) approach.

## 2. Materials and Methods

### 2.1. Bacterial Collection

A total of 14 bacterial strains included in this study were selected from a culture collection of isolates recovered from the inside and outside of the body of two different species of clinically healthy crickets: house cricket (**HC**) (*n* = 28), *A. domesticus*, and banded cricket (**BC**) (*n* = 54), *G. sigillatus*, during their rearing process before harvesting, at a local insect-rearing farm. The crickets were reared in plastic box containers with carton egg crates inside the boxes. The insects were fed with a dry plant powder and water. The boxes were located in a climate-controlled room. Live crickets were transported at room temperature in small plastic containers, with enough air for the insects, to the laboratory where the insects were euthanized by placing them at −20 °C briefly. Traditional cultivation methods using a direct plating approach on Columbia blood agar plates (Sigma Aldrich, Darmstadt, Germany) were performed with samples from the inside and outside of the insect body under aseptic conditions. Plates were incubated in aerobic conditions at 34 °C (house crickets) and 25 °C (banded crickets) according to the rearing temperature of the crickets for 24 h and up to 1 week. After the incubation period, colonies with macroscopy morphology differences were selected and transferred to fresh Columbia blood agar plates to obtain pure culture and subsequently identified at the species level by matrix-assisted laser desorption ionization time-of-flight mass spectrometry (MALDI-TOF MS) (Bruker Nederland BV, Leiderdorp, the Netherlands). The identified bacteria were preserved at −80 °C in buffer peptone water (BPW: Becton Dickinson GmbH, Heidelberg, Germany) with 20% glycerol. The list of the selected strains from **HC** and **BC** is shown in Table 1. In our selection, several members of the family *Enterobacteriaceae Staphylococcaceae*, *Enterococcaceae*, and *Bacillaceae* were included, all considered opportunistic pathogenic bacteria for humans and animals.

### 2.2. Genomic Characterization Using Whole Genome Sequencing

All the selected strains included in this study were molecularly characterized by a WGS approach. The selection was based on the following criteria: (a) opportunistic pathogenic bacteria for animals and humans, (b) potential entomopathogens detected in several crickets from the same species of the local farm, (c) clinically relevant strains of the ESKAPE pathogens—*Enterococcus faecium*, *Staphylococcus aureus*, *Klebsiella pneumoniae*, *Acinetobacter baumannii*, *Pseudomonas aeruginosa*, and *Enterobacter* spp. [27]. Since our main focus was to have a panoramic overview of the presence and diversity of resistance genes detected in crickets during their rearing process, our selection only included a single strain per insect species based on the criteria mentioned above.

Bacterial DNA was extracted using the Qiagen Blood and Tissue DNA isolation kit (Qiagen, Benelux B.V, Venlo, the Netherlands) from an overnight pure culture of each strain on heart infusion agar (HIS) (Becton Dickinson GmbH, Heidelberg, Germany) supplemented with 5% sheep blood, incubated at 37 °C for 24 h in aerobic conditions. DNA concentration was measured with a CLARIOstar Plus (BMG Labtech, Ortenberg, Germany). The isolated DNA was used for library preparation using the Illumina DNA Prep kit (Illumina, San Diego, CA, USA) according to the manufacturer’s instructions before sequencing on the Illumina MiSeq platform. DNA samples were sequenced based on a 300 bp paired-end read length on the Illumina Miseq sequencer (Illumina). Raw paired-end sequences were quality-checked using the FastQC tool (v.0.11.6), adaptors and low base quality scores below Q20 were filtered using bbduk from the BBMAP suite (v.38.98, https://jgi.doe.gov/data-and-tools/software-tools/bbtools/) accessed on 30 December 2024, followed by genome assembling using SPAdes (v.3.15.3) software [28]. QUAST v.5.0.2 [29] was used to evaluate the genome assembling quality. Checkm2 v1.0.2 [30] and BUSCO v5.5.0 [31] were implemented for the detection of potential contamination. The assembled draft genome was analyzed using star-amr (v. 0.7.2) software to determine the presence of antibiotic resistance genes (resfinder v.2.4.0), point mutations (point finder v.4.1.1), and the presence of plasmids (plasmidfinder v.2.2.0). Mobile genetic elements were investigated using MGEFinder v.1.0.3 and MGEdb v 1.0.2 [32]. The PubMed MLST V2.19.0 database tool was implemented to identify the sequence type (ST) belonging to the sequenced strains (https://pubmlst.org/) accessed 30 December 2024.

### 2.3. Accession Number

The assembled contigs/draft genomes for all the isolates characterized in this study have been deposited in the GenBank database with the accession numbers shown in Table 2. The GenBank BioProject ID is PRJNA1199941.

## 3. Results

In this study, the presence of AMR and disinfectant resistance genes was screened in a total of 14 selected culturable opportunistic pathogenic bacterial strains isolated from clinically healthy **HC** and **BC** (Table 1). The genomic information of the sequenced strains is described in Table 2. In total, 93% of the selected bacterial strains identified at the species level by the MALDI-TOF MS technique using the Bruker database included in the MALDI software (Bruker MALDI Biotyper® v.5.4.400.7052) were identified accurately and were comparable with the genomic information obtained from the sequenced strains. A misidentified strain (7%) member of the *E. cloacae* complex (ECC) initially identified by MALDI-TOF MS as *E. cloacae* was later identified as *Enterobacter hormaechei*, also a member of the ECC, by WGS.

**Table 2:** Genomic information of the 14 selected bacterial strains included in this study.

Source (Insect Species)	Strain Names (Label)	BioSample Accession	Bacterial Species	Number of Contigs (≥300 bp)	N50 Value	Genome Length (bp)	Coverage	GC %
House crickets	A_44	SAMN45893003	*Klebsiella pneumoniae*	217	56,825	5,097,926	90.4X	57.5
	A_43	SAMN45893004	*Staphylococcus warneri*	66	124,251	2,564,987	94X	32.56
	C_38	SAMN45893007	*Staphylococcus gallinarum*	52	102,590	2,822,451	102X	33.15
	C_41	SAMN45893008	*Staphylococcus arlettae*	57	116,579	2,698,571	86X	33.42
	C_49	SAMN45893009	*Mammaliicoccus sciuri*	60	169,743	3,012,826	91X	32.55
	B_57	SAMN45893006	*Enterococcus faecalis*	38	190,398	2,901,703	103X	37.29
	B_46	SAMN45893005	*Bacillus cereus*	201	105,960	6,081,965	79X	34.77
Banded crickets	A_20	SAMN45892996	*Klebsiella oxytoca*	218	76,125	6,089,801	97X	54.97
	A_31	SAMN45892999	*Klebisella pnuemoniae*	235	96,941	5,750,494	62X	56.51
	A_36	SAMN45893000	*Klebsiella aerogenes*	165	85,657	5,332,728	87X	54.69
	A_26	SAMN45892998	*Enterobacter hormaechei*	99	119,232	4,882,841	93X	55.28
	C_03	SAMN45893002	*Proteus mirabilis*	62	188,506	3,794,983	118X	38.53
	A_45	SAMN45893001	*Mammaliicoccus sciuri*	96	66,842	2,899,041	93X	32.41
	A_25	SAMN45892997	*Bacillus cereus*	79	170,596	5,657,686	105X	34.92

GC: Guanine and Cytosine.

The genome analysis using star-amr and ResFinder revealed the presence of several antibiotic resistance genes, the majority belonging to the antibiotic class β-lactams, followed by genes conferring resistance to phosphonic acid, lincosamide, phenicol, and tetracycline. The predicted phenotype, genotype, detected mobile genetic elements, plasmids, and sequence type of the 14 selected opportunist pathogenic bacterial strains investigated in this study are summarized in Table 3.

In **HC**, the presence of encoding genes conferring resistance to β-lactams was detected in a multidrug-resistant *K. pneumoniae* strain (A_44) carrying a broad-spectrum and an Extended-Spectrum β-Lactamase (ESBL) (*bla*_SHV-81_, *bla*_SHV-110_), in addition to the multidrug efflux pump (*Oqx*A, *Oqx*B); in this isolate, the presence of two plasmids, IncFIA(HI1) (identity: 98.45%, coverage: 99,74%) and IncFIB(K) (identity: 98.75%, coverage: 100%), and an ISKpn1 insertion element (identity: 99.7%, coverage: 100%) member of the IS3/IS150 transposases were identified.

Other encoding genes conferring resistance to β-lactams were identified in a facultative spore-forming bacterium, a *B. cereus* strain (B_46), carrying a β-lactam resistance gene (*bla*Z) harbored on a Col3M-like plasmid (identity: 98%, coverage: 100%) and an ISBce3 insertion element (identity: 99.5%, coverage: 100%) member of the IS200/IS605 transposases. Moreover, in both cricket species, a *mec*A1 gene, which is closely related to the *mec*A gene of Methicillin-Resistant *Staphylococcus aureus* (MRSA) and responsible for conferring resistance to methicillin, coding for penicillin-binding protein (PBP) 2A, was identified in *M. sciuri* strains (HC: C_49; BC: A_45).

Other resistance genes detected in **HC** correspond to a chromosomally located lincomycin resistance gene (*lsa*(A)) identified in one lactic acid bacterium (LAB), *E. faecalis*, belonging to ST638. A fosfomycin resistance gene, *fos*B1, a thiol transferase-encoding gene, member of the vicinal oxygen chelate (VOC) superfamily of metalloenzymes, was identified in *B. cereus* in both insect species (HC: B_46; BC: A_25). Both of these bacterial strains belong to clonal complex (CC) ST142, which is a dominant global *B. cereus* clonal complex, whereby in both bacterial strains the same insertion sequence (IS), ISBce3, was identified. In an *S. arlettae* (C_41) strain a disinfectant resistance gene belonging to the quaternary ammonium compounds (*qac*G) harbored in a rep13-like plasmid (identity: 99.89%, coverage: 100%) was detected.

In **BC**, the majority of the members of the *Enterobacteriaceae* family were carrying a β-lactamase gene in addition to other resistance genes, suggesting that β-lactamases are widely distributed and present in the opportunistic pathogenic bacterial species selected from this insect species. A multidrug-resistant *K. pneumoniae* strain (A_31) belonging to ST4975 carrying two chromosomally located ESBL genes, *bla*_SHV-70_ and *bla*_SHV-13_, in combination with a non-ESBL *bla*_SHV-11_ was identified. The presence of three insertion elements: ISKpn1 (identity: 99.9%, coverage: 100%) also detected in a *K. pneumoniae* (A_44) strain from **HC**, ISKpn28 (identity: 99.8%, coverage: 100%), a member of the IS481-like transposases, and ISKpn43 (identity: 99.8%, coverage: 100%), member of the IS110 transposase family, were detected in this strain. In addition, a fosfomycin (*fos*A) resistance gene and a multidrug efflux pump (*Oqx*A, *Oqx*B) were also detected. Moreover, in *K. oxytoca* ST525, a chromosomal class A ESBL (*bla*_OXY-2-6_) resistance gene was identified. A cephalosporin hydrolyzing class C β-lactamase (*bla*_ACT-16_) was detected in an *E. hormaechei* strain belonging to ST1955. Two plasmids, IncHI1A (identity: 100%, coverage: 100%) and IncHI1B (identity: 99%, coverage: 100%), and two insertion elements, ISEcl1, a member of the IS3 transposase family (identity: 99.9%, coverage: 100%), and IS5075, a member of the IS110 transposase family (identity: 99.7%, coverage: 100%), were identified in this strain.

Other AMR genes identified in **BC** correspond to chloramphenicol (*cat*) and tetracycline (*tet*(J)) genes identified in *P. mirabilis*.

No AMR genes or other resistance genes were identified in *Staphylococcus warneri*, *Staphylococcus gallinarum* from **HC**, and *Klebsiella aerogenes* from **BC**. In addition, no point mutations associated with AMR phenotypes were identified in the selected opportunistic pathogenic strains of this study.

## 4. Discussion

The selected opportunistic bacterial strains isolated from farmed crickets included in this study were characterized by WGS to identify the presence of AMR genes and mobile elements, providing a panoramic overview of the diversity of the AMR genes circulating during the cricket-rearing process. To the best of the authors’ knowledge, this report is the first to describe the occurrence of AMR genes and genetic mobile elements detected in live opportunistic pathogenic bacteria isolated during the rearing process from farm-reared **HC** and **BC,** using a WGS approach.

The most common AMR genes detected belong to the β-lactam class. These antimicrobial compounds are widely used for the control of infections in humans and animals. A scientific opinion published by the European Food Safety Authority (EFSA) in 2017 emphasized the need to increase farm biosecurity regarding the risk of ESBL and/or *Amp*C β-lactamases (*AmpC*) in food and in food-producing animals [33]. The results of our study confirm the presence of ESBL-producing bacteria in the insect production sector. ESBL-producing bacteria are present at least at low levels in all monitored primary production sectors in the Netherlands, and our current findings underline the need for a wider point-prevalence study in the insect production sector.

The most prevalent β-lactamase gene detected in our study corresponds to *bla*_SHV_; some ESBL-*bla*_SHV_ variants can occur naturally in *Enterobacteriaceae* such as *bla*_SHV-110_ and *bla*_SHV-13_, while others, e.g., *bla*_SHV-70_, are associated with acquired resistant to the 2be phenotype corresponding to ESBLs that hydrolyze third-generation cephalosporins [34]; particularly in ESBL-producing *K. pneumoniae*, the prevalence of these genes has been increasing worldwide [35]. Notably, these resistance genes have been previously identified mainly in clinical human isolates and livestock, frequently encoded by plasmids, highlighting the importance of the transmission and dissemination of antimicrobial resistance genes. The presence of other classes of β-lactamases was detected in *Klebsiella* and *Enterobacter*, both members of the *Enterobacteriaceae* family and part of the commensal intestinal microbiota of humans and animals. These species can also be found as spoiling bacteria in several environments including food, which highlights the risk of contamination. Mitchaothai et al. (2024) reported the presence of *Klebsiella* spp. and *Enterobacter* spp. during the production process of crickets and highlighted that drinking water could be a potential source of contamination via water pipes, representing a potential source of contamination with cricket frass [36].

Other β-lactam resistance genes detected correspond to the presence of the *mec*A gene detected in *M. sciuri* strains isolated from both cricket species in our study. The *mec*A gene has been previously identified in several species of marketed edible insects using bacterial DNA extracted from the insects [25] and in other habitats including humans, animals, and the environment [37]. Further studies on the relationship between the presence of *M. sciuri* in humans and animals could help to elucidate the risk and impact *of M. sciuri* colonization in edible crickets. Moreover, the presence of the *bla*Z gene detected in *B. cereus* in our study has been previously reported in ready-to-eat house crickets, mainly associated with other resistance genes detected by polymerase chain reaction (PCR) and nested PCR assays using bacterial DNA extracted directly from the insects [38]. In *Bacillus* spp., high prevalences of β-lactamases have been previously identified in humans, food, and environmental isolates [39], representing a public health concern due to their role as food-borne pathogens associated with gastrointestinal disease outbreaks in humans and food-related edible cricket products [40,41]. In insects, *Bacillus* spp. represent a potential risk for insect health, particularly when favorable conditions allow them to germinate and grow due to their particular characteristics to resist extreme conditions and chemical exposure [42]. In our study, the clonal complex detected in a *B. cereus* strain (CC142) represents a dominant clonal complex that could spread via the food chain and even between countries, highlighting the importance of global contamination risk assessment, monitoring, and safety management in the food production chain sector as reported by Tong et al., 2024 [43].

The second common AMR gene detected in both cricket species corresponds to *fos*A and *fos*B genes conferring resistance to fosfomycin, detected in *K. pneumoniae* and *B. cereus*, respectively. The fosfomycin resistance genes have been previously identified in pathogenic and non-pathogenic *B. cereus* isolates from foodstuffs [44]; however, according to the literature, this is the first study detecting the presence of phosphonic acid resistance genes (*fos*A, *fos*B1) in edible crickets.

Other AMR genes detected in these strains correspond to the tetracycline resistance gene tet(J) in *P. mirabilis* in **BC** and lincomycin resistance gene *lsa* (A) in *E. faecalis* in **HC**. Vandeweyer et al., 2019 [26] reported that the diversity of tetracycline resistance genes present in edible insects seems to be related to the insect-specific microbiome and insect feed. Enterococci inhabit the gastrointestinal tract of humans and several terrestrial animal species including invertebrates, insects, and mammals, and, due to their wide range of hosts, they can also be isolated from the environment inhabited by these hosts [45], whereby some species are used as probiotics. *E. faecalis* and *E. faecium* can cause human infections, particularly in hospitalized patients. In crickets, several Enterococci species have been identified during the rearing process [46] and have been associated with spoilage during storage [47]. Members of this genus are intrinsically resistant to lincosamides, aminoglycosides, streptogramins, and cephalosporins, which facilitates the acquisition of additional antibiotic resistance via horizontal gene transfer through mobile genetic elements, allowing Enterococci to coexist in close contact with other antibiotic-resistant bacteria and enhancing the transmission of AMR genes in their environment.

Interestingly, the presence of the multidrug efflux pump *oqx*AB genes detected in **BC** and **HC** in our study in *Enterobacteriaceae* has been previously reported in clinical human isolates as well as in food-producing animals, farmworkers, and the environment, suggesting that monitoring of the potential dissemination of these efflux pumps should be taken into consideration [48]. These efflux pumps play an important role in intrinsic and acquired AMR by decreasing the intracellular antibiotic concentration and facilitating the acquisition and spread of AMR via horizontal gene transfer. In *K. pneumoniae*, the efflux pump OqxAB, a member of the resistance-nodulation division (RND) family of efflux pumps, has been shown to promote β-lactam, chloramphenicol, and quinolone resistance [49].

A possible explanation for the presence of the *qac*G gene detected in Staphylococci could be the use of disinfectants, such as quaternary ammonium compounds used on the farm where the crickets were sampled. As reported by Donaghy et al. (2019) [50], the use of disinfectants in food production correlates positively with AMR development. The presence of disinfectant resistance genes can significantly harm the capacity of the disinfectant to act according to the manufacturer, particularly regarding the disinfection of bacteria carrying the *qac*G gene as previously described [51].

Regarding the presence of the mobile elements identified in this study, the *Inc*F conjugative replicon type detected in *K. pnuemoniae* (A_44) is one of the most common replicons identified in ESBL-harboring plasmids in *Enterobacteriaceae* from human and animal sources, playing an important role in the dissemination of ESBL genes; while the *Inc*H conjugative replicon type detected in *E. hormaechei* (A_26) has a wide host range including *Enterobacteriaceae* and also other Gram-negative bacteria originating from human, animal, and environmental sources. Moreover, the presence of the *Col*3M non-conjugative replicon type detected in a *B. cereus* strain carrying β-lactamase and fosfomycin resistance genes suggests the potential role of *Col*3M as a reservoir element for the acquisition of AMR genes. Further studies are needed to elucidate their potential dissemination role in the spread of AMR, particularly in the food industry sector. Likewise, the presence of several IS transposase families in the strains A_44 and B_46 from **HC** and in A_31, A_36, and A_25 from **BC** suggests a possible association with the mobilization and transfer of AMR genes that could contribute not only to the perpetuation and modification of the antibiotic-resistant phenotype but also the modulation of the pathogenic features of this opportunistic pathogenic bacterium.

According to the literature, a common group of five AMR genes belonging to tetracycline, macrolide, β-lactam, aminoglycosides, and vancomycin antibiotic classes with different levels of frequency and prevalence has been detected in husbandry animals where antibiotics are used as a prophylaxis method for the control of infections, indirectly contributing to the increase, accumulation, and dispersion of AMR in the farm environment [52]. In this context, insects can act as a reservoir and transmitter of AMR genes, thereby contributing to the AMR dissemination. In edible insect species such as mealworms (*Tenebrio molitor* and *Alphitobius diaperinus*) and crickets (*A. domesticus* and *G. sigillatus*), recent studies demonstrated the potential role of insect feed in the spread of AMR, suggesting that a thorough risk assessment analysis on AMR development and spread is needed during the insect-rearing process [26,53].

Therefore, good management practices according to Good Agricultural Practices (GAP) for cricket farms [54] should be implemented and a recommended monitoring for the detection of potential food safety hazards should be in place, including testing for AMR. The use of antibiotics should be avoided on insect farms, and disinfectants should be used with strict precautions, to avoid the selection of AMR and the development of resistance to disinfectants in bacteria, which has a potentially harmful effect on the reared cricket population and the animals and humans in the further feed and food chain.

## 5. Conclusions

To the best of the authors’ knowledge, this report represents the first attempt to describe the occurrence of AMR and other resistance genes in live opportunistic pathogenic bacteria isolated from farm-reared **HC** and **BC** during their rearing process, as well as the presence of mobile genetic elements by using a WGS approach. Despite the limited number of strains analyzed in this study, a pool of transferable AMR genes conferring resistance to mainly β-lactam and fosfomycin were detected in isolated bacteria from both crickets species, highlighting the risk of the potential spread of AMR genes via plasmids in bacteria between cricket batches and in the insect-rearing environment and their potential spread to livestock and humans via food and feed. Further research is essential to unravel the source of these AMR genes and their transmission dynamics during the insect-rearing process, considering the increasing use and importance of edible insects as a modern protein source in the feed and food production chain.

## Figures and Tables

**Table 1:** Bacterial strains isolated from farm-reared house crickets (*Acheta domesticus*) and banded crickets (*Gryllodes sigillatus*). List of selected strains for this study.

Source (Insect Species)	Taxon (Family)	Strain Names (Label)	Bacterial Species
House crickets	*Enterobacteriaceae*	A_44	*Klebsiella pneumoniae*
	*Staphylococcaceae*	A_43	*Staphylococcus warneri*
		C_38	*Staphylococcus gallinarum*
		C_41	*Staphylococcus arlettae*
		C_49	*Mammaliicoccus sciuri*
	*Enterococcaceae*	B_57	*Enterococcus faecalis*
	*Bacillaceae*	B_46	*Bacillus cereus*
Banded crickets	*Enterobacteriaceae*	A_20	*Klebsiella oxytoca*
		A_31	*Klebsiella pneumoniae*
		A_36	*Klebsiella aerogenes*
		A_26	*Enterobacter cloacae*
		C_03	*Proteus mirabilis*
	*Staphylococcaceae*	A_45	*Mammaliicoccus sciuri*
	*Bacillaceae*	A_25	*Bacillus cereus*

**Table 3:** Overview of the predicted phenotype, genotype, and mobile elements identified in the sequenced bacterial strains from house crickets (*Acheta domesticus*) and banded crickets (*Gryllodes sigillatus*).

Source (Insect Species)	Strain Names (Label)	Bacterial Species	Predicted Phenotype	Genotype	Plasmid	MGEs	Sequence Type
House crickets	A_44	*Klebsiella pneumoniae*	AMO, AMP, PIP, TIC	*blaSHV-81*	IncFIA(HI1), IncFIB(K)	ISKpn1	Unknown
			Unknown beta-lactam	*blaSHV-110*			
			CHL, NAL, CIP, TRI, benzalkonium chloride, cetylpyridinium chloride	*OqxA, OqxB*			
	A_43	*Staphylococcus warneri*	-	No detected	None detected	None detected	-
	C_38	*Staphylococcus gallinarum*	-	No detected	None detected	None detected	-
	C_41	*Staphylococcus arlettae*	chlorhexidine, benzalkonium chloride, ethidium bromide, cetylpyridinium chloride	*qac*G	rep13	None detected	-
	C_49	*Mammaliicoccus sciuri*	AMC, PIT, AMP, CXI, CTZ, CEP, PIP, CTA, MEM, AMO, CIX, IMI, ERT	*mec*A1	None detected	None detected	-
	B_57	*Enterococcus faecalis*	Lincomycin	*lsa*(A)	None detected	None detected	638
	B_46	*Bacillus cereus*	FOS	*fos*B1	Col3M	ISBce3	1662 CC ST-142
			AMO, AMP, PEN, PIP	*bla*Z			
Banded crickets	A_20	*Klebsiella oxytoca*	CTA, TIC, AMP, CTZ, PIT, CXI, PIP, AMO, AMC, TIL	*blaOXY*-2-6	None detected	None detected	525
	A_31	*Klebsiella pneumoniae*	TIC, AMP, PEN, CTA, AZT, CTR, CTZ, AMO, CEP	*blaSHV-70*	None detected	ISKpn1, ISKpn28, ISKpn43	4975
			TIC, AMP, PIP, AMO	*blaSHV-11*			
			TIC, AMP, PEN, CTA, AZT, CTR, CTZ, AMO, CEP	*blaSHV-13*			
			FOS	*fos*A			
			CHL, NAL, CIP, TRI, cetylpyridinium chloride, benzalkonium chloride	*Oqx*A, *Oqx*B			
	A_36	*Klebsiella aerogenes*	-	No detected	None detected	None detected	-
	A_26	*Enterobacter hormaechei*	TIC, AMO, CTZ, TIL, PIP, PIT, AMO, AMP, CTA	blaACT-16	IncHI1A, IncHI1B	ISEcI1, IS5075	1955
	C_03	*Proteus mirabilis*	CHL	*cat*	None detected	None detected	-
			TET	*tet(J)*			
	A_45	*Mammaliicoccus sciuri*	AMC, AMP, MEM, AMO, PIP, IMI, ERT, PIT, CTA, CEP, CIX, CXI, CTZ	*mec*A1	None detected	None detected	-
	A_25	*Bacillus cereus*	FOS	*fos*B1	None detected	ISBce3	CC ST 142

Legend: Amoxicillin (AMO), Ampicillin (AMP), Piperacillin (PIP), Ticarcillin (TIC), Chloramphenicol (CHL), Nalidixic acid (NAL), Ciprofloxacin (CIP), Trimethoprim (TRI), Amoxicillin–clavulanic acid (AMC), Piperacillin–tazobactam (PIT), Cefixime (CIX), Ceftazidime (CTZ), Cefepime (CEP), Cefotaxime (CTA), Meropenem (MEM), Cefoxitin (CXI), Imipenem (IMI), Ertapenem (ERT), Fosfomycin (FOS), Penicillin (PEN), Ticarcillin–clavulanate (TIL), Aztreonam (AZT), Ceftriaxone (CTR), Tetracycline (TET).

## Data Availability

The assembled contigs/draft genomes for all the isolates characterized in this study have been deposited in the GenBank database.

## References

[1] Van Huis A., Van Itterbeeck J., Klunder H., Merterns E., Halloran A., Muir G., Vantomme P. (2013). Edible Insects Future Prospects for Food and Feed Security.

[2] UN DESA Population Division (2024). Population Division World Population Prospects 2024 Data Sources.

[3] van der Spiegel M., Noordam M.Y., van der Fels-Klerx H.J. (2013). Safety of Novel Protein Sources (Insects, Microalgae, Seaweed, Duckweed, and Rapeseed) and Legislative Aspects for Their Application in Food and Feed Production. Compr. Rev. Food Sci. Food Saf..

[4] van Huis A. (2016). Edible Insects Are the Future?. Proc. Nutr. Soc..

[5] Belluco S., Losasso C., Maggioletti M., Alonzi C.C., Paoletti M.G., Ricci A. (2013). Edible Insects in a Food Safety and Nutritional Perspective: A Critical Review. Compr. Rev. Food Sci. Food Saf..

[6] Schlüter O., Rumpold B., Holzhauser T., Roth A., Vogel R.F., Quasigroch W., Vogel S., Heinz V., Jäger H., Bandick N. (2017). Safety Aspects of the Production of Foods and Food Ingredients from Insects. Mol. Nutr. Food Res..

[7] Lange K.W., Nakamura Y. (2023). Potential Contribution of Edible Insects to Sustainable Consumption and Production. Front. Sustain..

[8] (2015). REGULATION (EU) 2015/2283 of the European Parliament and of the Council—of 25 November 2015—on Novel Foods, Amending Regulation (EU) No 1169/2011 of the European Parliament and of the Council and Repealing Regulation (EC) No 258/97 of the European Parliament and of the Council and Commission Regulation (EC) No 1852/2001.

[9] (2015). Risk Profile Related to Production and Consumption of Insects as Food and Feed. EFSA J..

[10] European Commission Commission Regulation (EU) 2021/1925 of 5 November 2021 Amending Certain Annexes to Regulation (EU) No 142/2011 as Regards the Requirements for Placing on the Market of Certain Insect Products and the Adaptation of a Containment Method. https://eur-lex.europa.eu/legal-content/en/TXT/?uri=CELEX:32021R1925&qid=1727827312390.

[11] European Commission (2017). Commission Implementing Regulation (EU) 2017/2470—of 20 December 2017—Establishing the Union List of Novel Foods in Accordance with Regulation (EU) 2015/2283 of the European Parliament and of the Council on Novel Foods.

[12] European Commission (EU) (2023). Commission Implementing Regulation (EU) 2023/5 of 3 January 2023 Authorising the Placing on the Market of Acheta Domesticus (House Cricket) Partially Defatted Powder as a Novel Food and Amending Implementing Regulation (EU) 2017/2470.

[13] Magara H.J.O., Niassy S., Ayieko M.A., Mukundamago M., Egonyu J.P., Tanga C.M., Kimathi E.K., Ongere J.O., Fiaboe K.K.M., Hugel S. (2021). Edible Crickets (Orthoptera) Around the World: Distribution, Nutritional Value, and Other Benefits—A Review. Front. Nutr..

[14] Hanboonsong A., Durst P. (2020). Guidance on Sustainable Cricket Farming—A Practical Manual for Farmers and Inspectors.

[15] Kemsawasd V., Inthachat W., Suttisansanee U., Temviriyanukul P. (2022). Road to The Red Carpet of Edible Crickets through Integration into the Human Food Chain with Biofunctions and Sustainability: A Review. Int. J. Mol. Sci..

[16] Hoek-van den Hil E.F., Antonis A.F.G., Brouwer M.S.M., Bruins M.E., Dame M.A., van Groenestijn J.W., Haenen O.L.M., Hoffmans Y., Meijer N.P., Veldkamp T. (2022). Use of Insects for Food and Feed: Scientific Overview of the Present Knowledge on Insect Rearing, Use of Residual Streams as Substrates, and Safety Aspects.

[17] Ng S.H., Stat M., Bunce M., Simmons L.W. (2018). The Influence of Diet and Environment on the Gut Microbial Community of Field Crickets. Ecol. Evol..

[18] Hosokawa T., Kikuchi Y., Fukatsu T. (2007). How Many Symbionts Are Provided by Mothers, Acquired by Offspring, and Needed for Successful Vertical Transmission in an Obligate Insect–Bacterium Mutualism?. Mol. Ecol..

[19] van der Fels-Klerx H.J., Camenzuli L., Belluco S., Meijer N., Ricci A. (2018). Food Safety Issues Related to Uses of Insects for Feeds and Foods. Compr. Rev. Food Sci. Food Saf..

[20] Wang H., Mc Entire J.C., Zhang L., Li X., Doyle M.P. (2012). The Transfer of Antibiotic Resistance from Food to Humans: Facts, Implications and Future Directions. Revue Scientifique et Technique de l’OIE.

[21] Council on Animal Affairs (RDA) (2018). The Emerging Insect Industry.

[22] Vandeweyer D. (2018). Microbiological Quality of Raw Edible Insects and Impact of Processing and Preservation.

[23] Vandeweyer D., De Smet J., Van Looveren N., Van Campenhout L. (2021). Biological Contaminants in Insects as Food and Feed. J. Insects Food Feed.

[24] Zurek L., Ghosh A. (2014). Insects Represent a Link between Food Animal Farms and the Urban Environment for Antibiotic Resistance Traits. Appl. Environ. Microbiol..

[25] Milanović V., Osimani A., Pasquini M., Aquilanti L., Garofalo C., Taccari M., Cardinali F., Riolo P., Clementi F. (2016). Getting Insight into the Prevalence of Antibiotic Resistance Genes in Specimens of Marketed Edible Insects. Int. J. Food Microbiol..

[26] Vandeweyer D., Milanović V., Garofalo C., Osimani A., Clementi F., Van Campenhout L., Aquilanti L. (2019). Real-Time PCR Detection and Quantification of Selected Transferable Antibiotic Resistance Genes in Fresh Edible Insects from Belgium and the Netherlands. Int. J. Food Microbiol..

[27] Miller W.R., Arias C.A. (2024). ESKAPE Pathogens: Antimicrobial Resistance, Epidemiology, Clinical Impact and Therapeutics. Nat. Rev. Microbiol..

[28] Prjibelski A., Antipov D., Meleshko D., Lapidus A., Korobeynikov A. (2020). Using SPAdes De Novo Assembler. Curr. Protoc. Bioinform..

[29] Gurevich A., Saveliev V., Vyahhi N., Tesler G. (2013). QUAST: Quality Assessment Tool for Genome Assemblies. Bioinformatics.

[30] Chklovski A., Parks D.H., Woodcroft B.J., Tyson G.W. (2023). CheckM2: A Rapid, Scalable and Accurate Tool for Assessing Microbial Genome Quality Using Machine Learning. Nat. Methods.

[31] Manni M., Berkeley M.R., Seppey M., Simão F.A., Zdobnov E.M. (2021). BUSCO Update: Novel and Streamlined Workflows along with Broader and Deeper Phylogenetic Coverage for Scoring of Eukaryotic, Prokaryotic, and Viral Genomes. Mol. Biol. Evol..

[32] Johansson M.H.K., Bortolaia V., Tansirichaiya S., Aarestrup F.M., Roberts A.P., Petersen T.N. (2021). Detection of Mobile Genetic Elements Associated with Antibiotic Resistance in *Salmonella enterica* Using a Newly Developed Web Tool: MobileElementFinder. J. Antimicrob. Chemother..

[33] (2017). ECDC, EFSA and EMA Joint Scientific Opinion on a List of Outcome Indicators as Regards Surveillance of Antimicrobial Resistance and Antimicrobial Consumption in Humans and Food-producing Animals. EFSA J..

[34] Naas T., Oueslati S., Bonnin R.A., Dabos M.L., Zavala A., Dortet L., Retailleau P., Iorga B.I. (2017). Beta-Lactamase DataBase (BLDB)—Structure and Function. J. Enzyme Inhin. Med. Chem..

[35] Neubauer S., Madzgalla S., Marquet M., Klabunde A., Büttner B., Göhring A., Brandt C., Feller K.-H., Pletz M.W., Makarewicz O. (2020). A Genotype-Phenotype Correlation Study of SHV β-Lactamases Offers New Insight into SHV Resistance Profiles. Antimicrob. Agents Chemother..

[36] Mitchaothai J., Grabowski N.T., Lertpatarakomol R., Trairatapiwan T., Lukkananukool A. (2024). Bacterial Contamination and Antimicrobial Resistance in Two-Spotted (Gryllus Bimaculatus) and House (Acheta Domesticus) Cricket Rearing and Harvesting Processes. Vet. Sci..

[37] Nemeghaire S., Argudín M.A., Feßler A.T., Hauschild T., Schwarz S., Butaye P. (2014). The Ecological Importance of the Staphylococcus Sciuri Species Group as a Reservoir for Resistance and Virulence Genes. Vet. Microbiol..

[38] Roncolini A., Cardinali F., Aquilanti L., Milanović V., Garofalo C., Sabbatini R., Abaker M.S.S., Pandolfi M., Pasquini M., Tavoletti S. (2019). Investigating Antibiotic Resistance Genes in Marketed Ready-to-Eat Small Crickets (*Acheta domesticus*). J. Food Sci..

[39] Savic D., Miljkovic-Selimovic B., Lepsanovic Z., Tambur Z., Konstantinovic S., Stankovic N., Ristanovic E. (2016). Antimicrobial Susceptibility and β-Lactamase Production in Bacillus Cereus Isolates from Stool of Patients, Food and Environment Samples. Vojnosanit. Pregl..

[40] Bottone E.J. (2010). Bacillus Cereus, a Volatile Human Pathogen. Clin. Microbiol. Rev..

[41] Fasolato L., Cardazzo B., Carraro L., Fontana F., Novelli E., Balzan S. (2018). Edible Processed Insects from E-Commerce: Food Safety with a Focus on the Bacillus Cereus Group. Food Microbiol..

[42] Ter Beek A., Brul S. (2010). To Kill or Not to Kill Bacilli: Opportunities for Food Biotechnology. Curr. Opin. Biotechnol..

[43] Tong C., Xiao D., Li Q., Gou J., Wang S., Zeng Z., Xiong W. (2024). First Insights into the Prevalence, Genetic Characteristics, and Pathogenicity of *Bacillus cereus* from Generations Worldwide. mSphere.

[44] Sornchuer P., Saninjuk K., Amonyingcharoen S., Ruangtong J., Thongsepee N., Martviset P., Chantree P., Sangpairoj K. (2024). Whole Genome Sequencing Reveals Antimicrobial Resistance and Virulence Genes of Both Pathogenic and Non-Pathogenic B. Cereus Group Isolates from Foodstuffs in Thailand. Antibiotics.

[45] Fiore E., Van Tyne D., Gilmore M.S. (2019). Pathogenicity of Enterococci. Microbiol. Spectr..

[46] Garofalo C., Osimani A., Milanović V., Taccari M., Cardinali F., Aquilanti L., Riolo P., Ruschioni S., Isidoro N., Clementi F. (2017). The Microbiota of Marketed Processed Edible Insects as Revealed by High-Throughput Sequencing. Food Microbiol..

[47] Channaiah L.H., Subramanyam B., McKinney L.J., Zurek L. (2010). Stored-Product Insects Carry Antibiotic-Resistant and Potentially Virulent Enterococci. FEMS Microbiol. Ecol..

[48] Zhao J., Chen Z., Chen S., Deng Y., Liu Y., Tian W., Huang X., Wu C., Sun Y., Sun Y. (2010). Prevalence and Dissemination of *OqxAB* in *Escherichia coli* Isolates from Animals, Farmworkers, and the Environment. Antimicrob. Agents Chemother..

[49] Razavi S., Mirnejad R., Babapour E. (2020). Involvement of AcrAB and OqxAB Efflux Pumps in Antimicrobial Resistance of Clinical Isolates of *Klebsiella pneumonia*. J. Appl. Biotechnol.Rep..

[50] Donaghy J.A., Jagadeesan B., Goodburn K., Grunwald L., Jensen O.N., Jespers A., Kanagachandran K., Lafforgue H., Seefelder W., Quentin M.-C. (2019). Relationship of Sanitizers, Disinfectants, and Cleaning Agents with Antimicrobial Resistance. J. Food Prot..

[51] Walker M.A., Singh A., Gibson T.W., Rousseau J., Weese J.S. (2020). Presence of Qac Genes in Clinical Isolates of Methicillin-resistant and Methicillin-susceptible *Staphylococcus pseudintermedius* and Their Impact on Chlorhexidine Digluconate Susceptibility. Vet. Surg..

[52] Raka R.N., Zhang L., Chen R., Xue X. (2024). Antibiotic Resistance Genes in Global Food Transformation System: Edible Insects vs. Livestock. Foods.

[53] Osimani A., Milanović V., Cardinali F., Garofalo C., Clementi F., Ruschioni S., Riolo P., Isidoro N., Loreto N., Galarini R. (2018). Distribution of Transferable Antibiotic Resistance Genes in Laboratory-Reared Edible Mealworms (*Tenebrio molitor* L.). Front. Microbiol..

[54] National Bureau of Agricultural Commodity and Food Standards, Ministry of Agriculture and Cooperatives (2017). Thai Agricultural Standard (TAS 8202-2017): Good Agricultural Practices for Cricket Farm.

